# Disappearance of bilateral essential tremor after acute stroke: A case report and literature review

**DOI:** 10.1097/MD.0000000000043537

**Published:** 2026-01-23

**Authors:** Linjing Song, Dayong Ma, Hua Zhang, Jingpei Wei, Chao Zhang, Haihuan Yang, Ruiyun Yu, Xiaocheng Wang, Yuanyuan Wang, Li Wang, Baoyun Qi

**Affiliations:** a Dongzhimen Hospital, Beijing University of Chinese Medicine, Beijing, China.

**Keywords:** acute stroke, essential tremor, nerve fiber tracking

## Abstract

**Rationale:**

Acute ischemic stroke is an important cause of in worldwide mortality and morbidity. nevertheless, in certain instances of concomitant idiopathic tremor, stroke lesions may have a therapeutic effect in alleviating the symptoms of idiopathic tremor. Limited reports exist on the improvement of essential tremor (ET) following a stroke. This research presents a case of symptom improvement in ET following acute stroke thrombolysis in the right centrum semiovale of a patient.

**Patient concerns:**

The patient is a retired 75-year-old male who is right-handed. He was hospitalized on January 1, 2021, due to limited movement in his left limbs and difficulties with speech. The patient has experienced a bilateral tremor in the upper limbs for 30 years, with exacerbation on the left side. The left finger-nose test lacked stability and precision.

**Diagnoses:**

The cranial computed tomography revealed scattered lacunar infarctions in the brain. The magnetic resonance imaging revealed many lacunar lesions in the bilateral basal ganglia and the centrum semiovale. A fresh lesion was seen in the right centrum semiovale.

**Outcomes:**

In the treatment of cerebral infarction, patients did not receive treatment and intervention for tremor symptoms. However, the patient also showed significant improvement in tremor in both upper extremities after the symptoms of acute cerebral infarction subsided, and the finger–nose test was consistent and precise on both sides. Fahn-Tolosa-Marin tremor rating scale 7 points.

**Lessons:**

The three-dimensional T1-weighted imaging, diffusion weighted imaging and diffusion tensor imaging sequences of magnetic resonance imaging images were analyzed, and BrainVoyager was used to visualize the location relationship between the infarct focus, thalamus and related fiber conduction tracts. It was inferred that the descending corticospinal tract and the 2 ascending conduction tracts from thalamus to cerebral cortex formed a complete neural circuit. The location of the infarct intersects the above conduction tract and thus interferes with the oscillatory network within it. Therefore, it is possible to break the oscillation network of ET by interfering with a certain node, which can provide more ideas for the treatment of ET in the future.

## 1. Introduction

Essential tremor (ET) is the most common movement disorder, characterized by action tremor in both upper limbs, which is particularly noticeable during daily activities such as writing, pouring water, and eating. The tremor may also affect the lower limbs, head, mouth, face, or voice.^[1]^ In addition to motor symptoms, ET patients may also experience sensory impairments, psychiatric symptoms, sleep disorders, and other non-motor symptoms, which often have a hereditary tendency. Although the severity of tremor is not related to mortality, studies have shown that ET patients have a higher incidence of Parkinson’s disease than normal people, and some patients may have difficulty in daily living due to severe tremors.^[2]^ In the past nearly 30 years, some researchers have reported cases of ET symptoms disappearing in poststroke patients, and the following case report and literature review will further explore the pathological mechanism of ET.

## 2. The relevant details of the case

### 2.1. Case introduction

**Figure 1.**
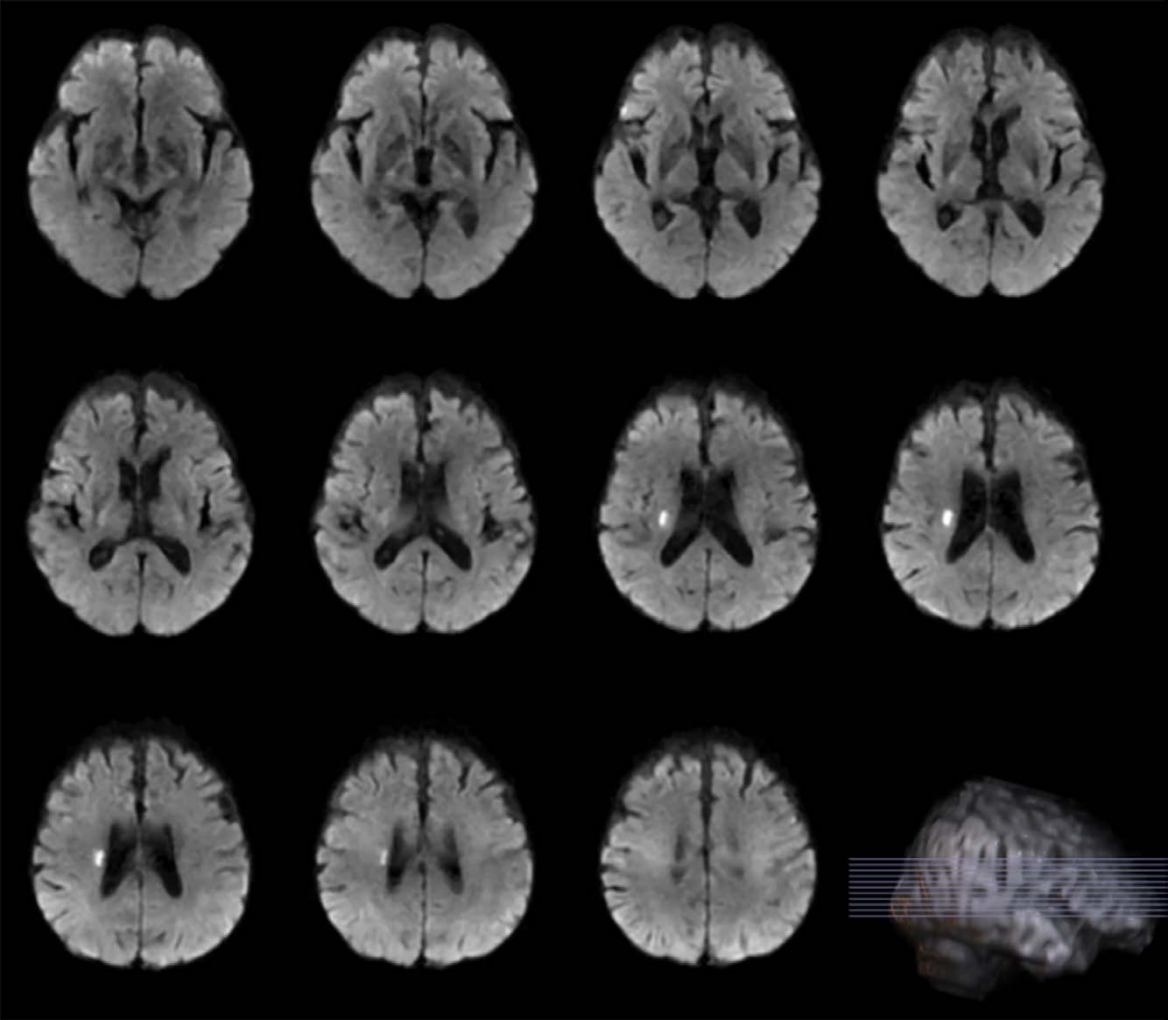
New infarct foci in the center of the right centrum semiovale of the patient’s brain The white highlights shown in the figure are infarct ischemic foci.

The patient is a retired 75-year-old male who is right-handed. He was hospitalized on January 1, 2021, due to limited movement in his left limbs and difficulties with speech. The cranial computed tomography revealed scattered lacunar infarctions in the brain. The magnetic resonance imaging (MRI) revealed many lacunar lesions in the bilateral basal ganglia and the centrum semiovale. A fresh lesion was seen in the right centrum semiovale (Fig. 1). The patient has experienced a bilateral tremor in the upper limbs for 30 years, with exacerbation on the left side. The left finger–nose test lacked stability and precision. He has a medical history of constipation, hemorrhoids, and hyperlipidemia. However, he has no prior history of hypertension, coronary artery disease, or diabetes mellitus. The family history indicates that the patient has 2 sons, both of whom exhibit a tendency for tremor. Following intravenous thrombolytic therapy for acute cerebral infarction, the patient’s left limb mobility and speech difficulties improved compared to prior conditions. Simultaneously, the tremor in both upper limbs exhibited substantial improvement, and the finger–nose tests on either side were consistent and precise. Fahn-Tolosa-Marin tremor rating scale Score 7 (Fig. 1).

### 2.2. Image analysis of MRI

**Figure 2.**
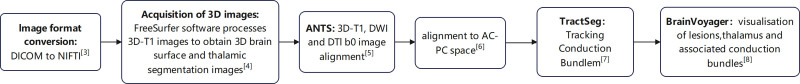
MRI image analysis process. 3D-T1 = three-dimensional T1 weighted imaging, AC = anterior commissure, ANTS = advanced normalization tools, DICOM = digital imaging and communications in medicine, DTI = diffusion tensor imaging, DWI = diffusion weighted imaging, MRI = magnetic resonance imaging, NIFTI = neuroimaging informatics technology initiative, PC = posterior commissure.

**Figure 3.**
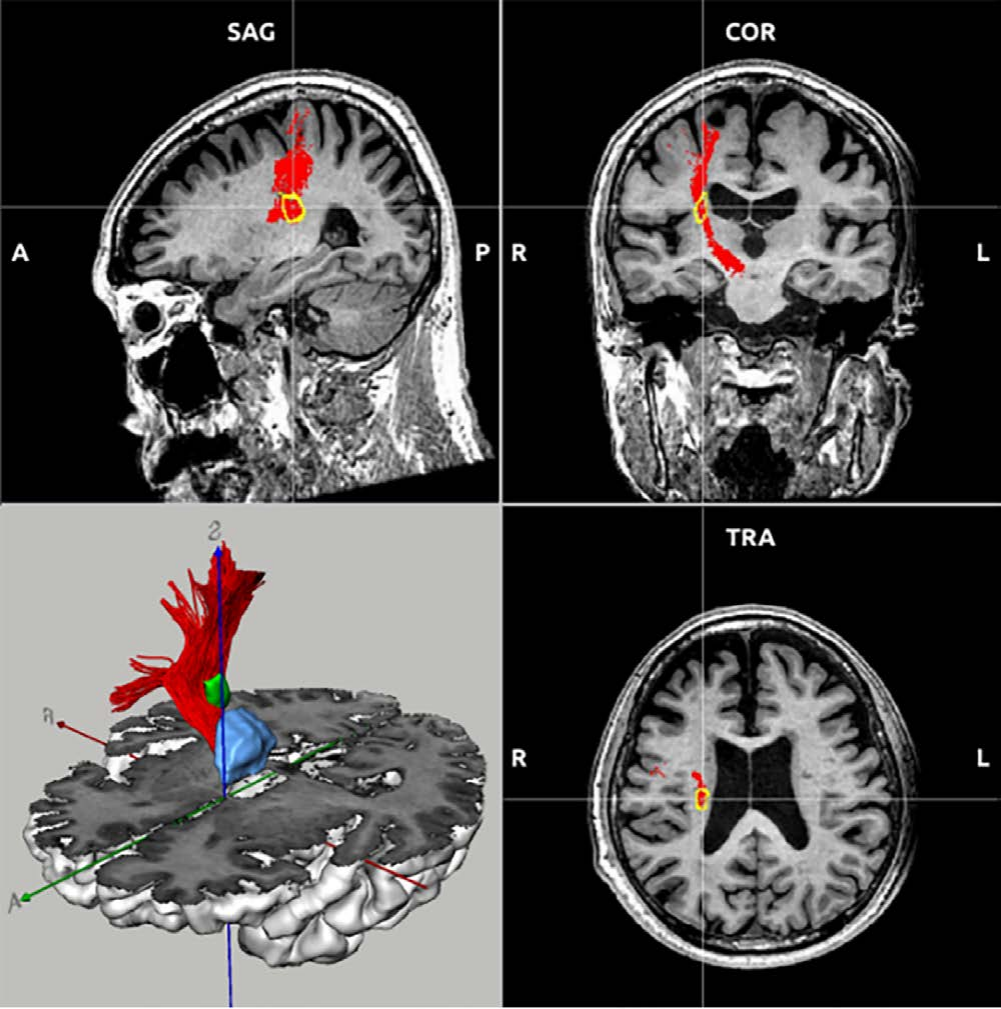
The infarct focus (yellow circle, green) intersects the location of the corticospinal tract (red) and thalamus (blue) in the brain. COR = coronal, SAG = sagittal, TRA = transverse.

**Figure 4.**
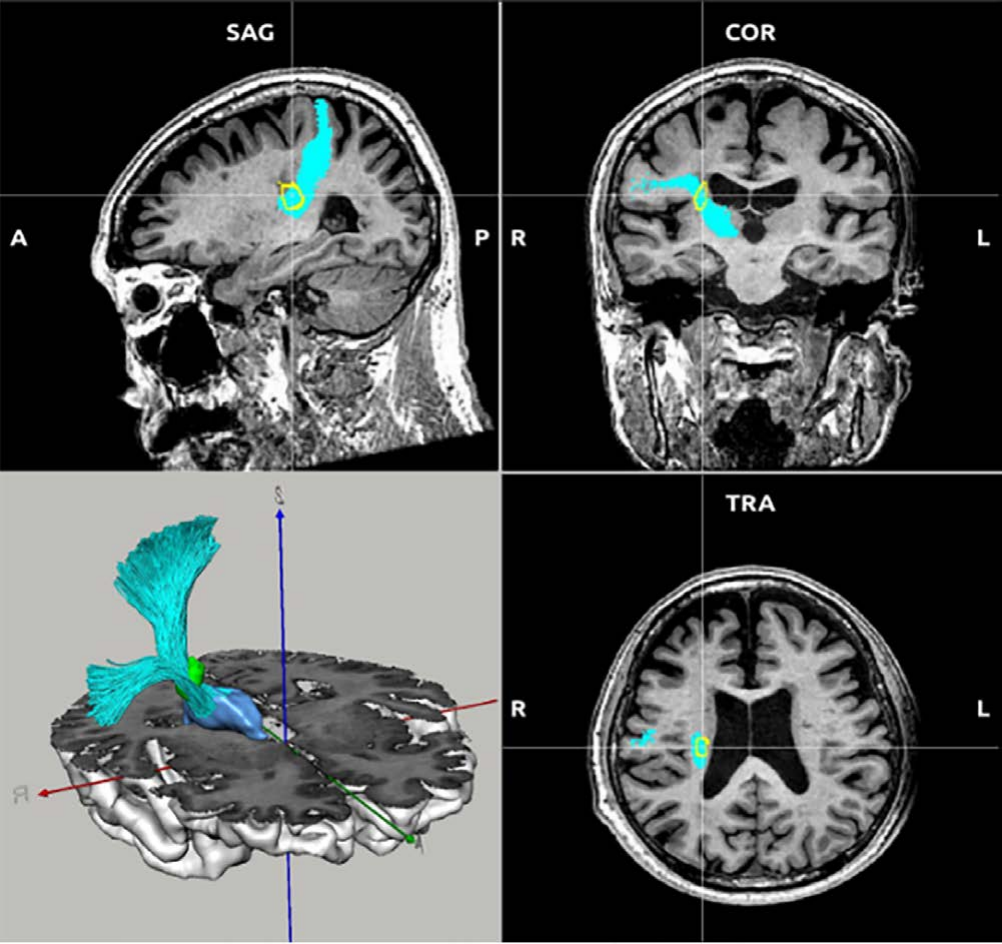
Infarct foci (yellow circle, green) intersect with thalamo-postcentral gyrus fibers (T_POSTC; cyan) and thalamus (blue) in the brain. COR = coronal, SAG = sagittal, TRA =transverse.

**Figure 5.**
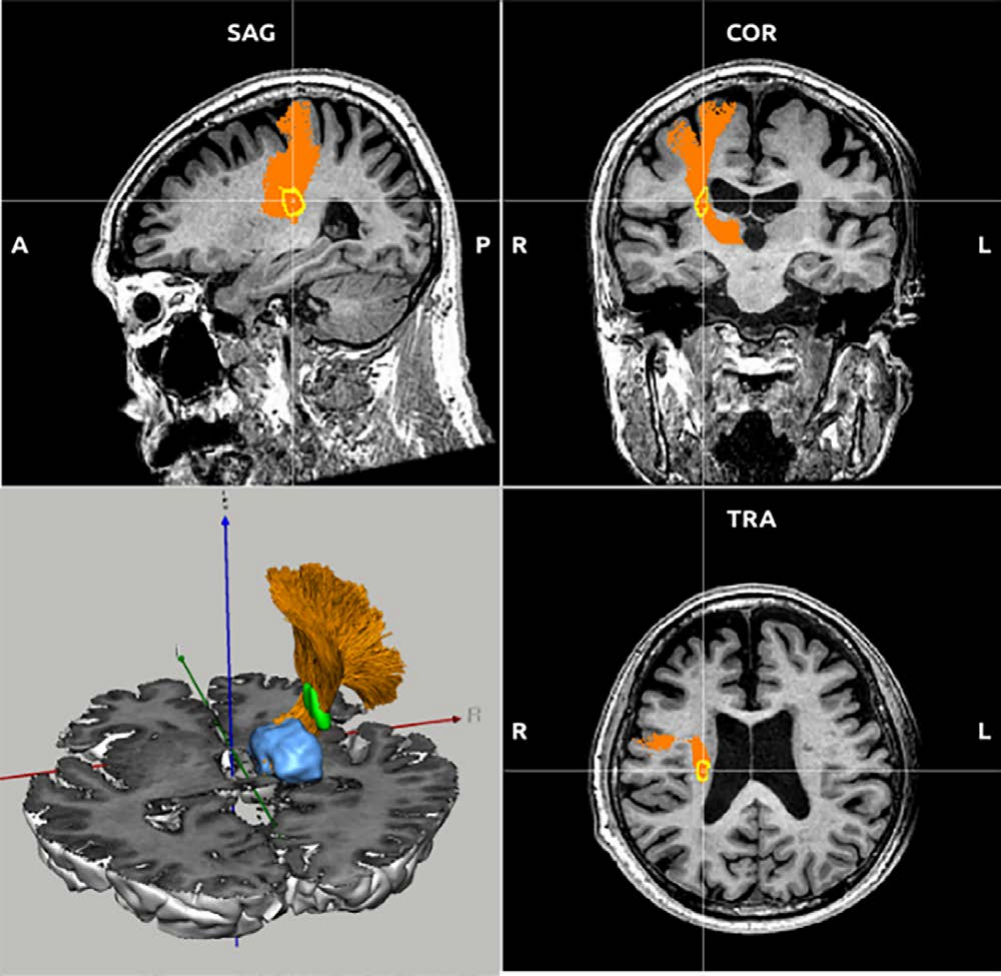
Infarct foci (yellow circle, green) intersect with thalamo-precentral gyrus fibers (T_PREC; orange), thalamus (blue) in the brain. COR = coronal, SAG = sagittal, TRA = transverse.

**Figure 6.**
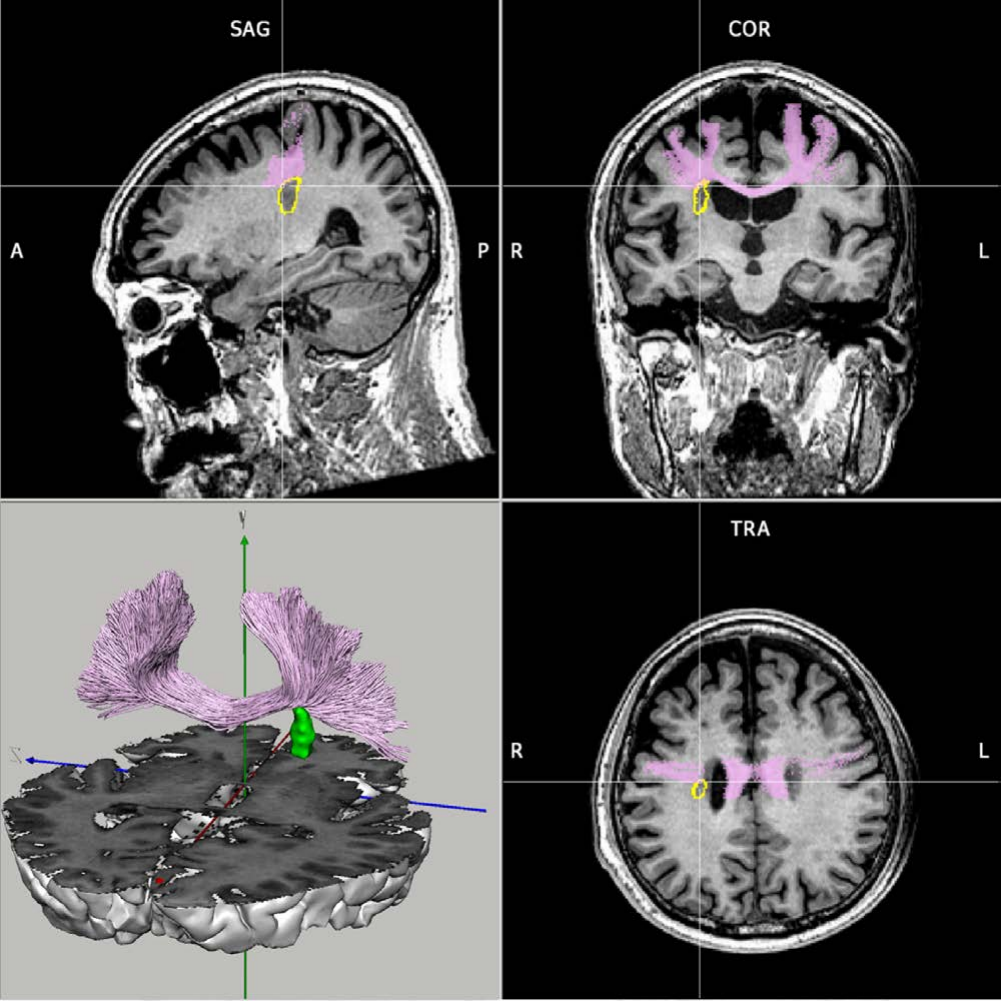
Location information of the infarcted site (yellow circle, green) and corpus callosum (purple) in the brain. COR = coronal, SAG = sagittal, TRA = transverse.

[1] Three-dimensional T1-weighted imaging:TR = 7.95 ms, TE = 3.07 ms, matrix = 512 × 512, field of view (FOV) = 240 × 240 mm, slice thickness = 1.0 mm;
[2] Diffusion weighted imaging:TR = 5000.0 ms, TE = 93.5 ms, matrix = 256 × 256, FOV = 240 × 240 mm, slice thickness = 7.0 mm;
[3] Diffusion tensor imaging:TR = 11,000.0 ms, TE = 94 ms, matrix = 256 × 256, FOV = 240 × 240 mm, slice thickness = 2.9 mm, *b*-value = 1000, number of directions = 30, number of *b*_0_ = 1^[3–8]^ (Figs. 2–6).

## 3. Discussion

### 3.1. The pathogenesis of ET

In line with the findings of the literature study (Table 1),^[9–21]^ the majority of ET patients (between 30% and 70%) have a family history, and the condition most frequently affects middle aged and older persons over the age of 40. Oscillatory networks in the brain are believed to significantly contribute to the etiology of ET. Numerous regions of the brain, including the cerebellum, thalamus, olivary nucleus, and cortical motor areas, participate in the initiation of ET oscillations.^[22]^ A thorough review of the literature indicates that the oscillatory network associated with ET may involve significant neural conduction nodes such as the cerebral cortex, centrum semiovale, corona radiata, posterior limb of the internal capsule, striatum, thalamus, pons, and cerebellum (Table 1).^[9–21]^ The olivocerebellar system and thalamus are pivotal structures, while cortical motor regions are periodically synchronized with tremor rhythms in thalamocortical circuits; tremor is not produced by a singular oscillator; dynamic alterations in network composition and interactions within symmetrical loops are characteristic of tremor generation.^[23]^ The cerebello-thalamo-cortical loop is posited to be pivotal, whereas neurotransmitters like γ-aminobutyric acid and glutamate are also recognized as significant contributors to the cerebello-thalamo-cortical circuit^[24],[9–21]^ (Table 1).

**Table 1:** Summary of characteristics of previous infarction cases.

Location of infarction	Literature sources	Specific infarct location	Age	Sex	Symptoms of ET	Family medical history	Improvement in ET
Cortical and subcortical infarcts	Joong-Seok Kim et al^[9]^	The left precentral gyrus of the frontal cortex experienced infarction.	63	Female	Tremor in both hands for 5 yr, significant on the left side, with head tremor	Yes	Right sided tremor disappeared, left hand and head tremor significantly improved
	Anne E.A. et al^[10]^	Infarction of the right frontal cortex	75	Male	Mild bilateral tremor since 45 yr of age	Yes	Left arm tremor disappeared, right arm tremor worsened
	Michel J.M. et al^[11]^	Cortical-subcortical and precortical infarcts	1948s	Male	Tremor in both hands for over 10 yr	NA	Recurrence of left-sided tremor 3 mo after its disappearance
	Michel J.M. et al^[11]^	Numerous minor subcortical hemorrhages were present (<10 mm)	1933s	Female	Tremor in both hands for 10 yr	NA	Loss of tremor in the left limb
	Moussa A. Chalah et al^[12]^	Right cortical and subcortical anterior central gyrus infarction	76	Female	Tremor of both upper limbs involving the head and voice at 56 yr of age	No	Loss of tremor in the left upper limb
Infarction of the white matter of the brain	F. Le Pira et al^[13]^	Small lesion in the left corona radiata	73	Male	Tremor of the limbs	Yes	Loss of tremor in the right upper limb
	Chunhua Xi et al^[14]^	Left radial coronary infarction	83	Male	Bilateral postural tremor of the limbs for more than 60 yr	Yes	Loss of tremor in the right upper and lower limbs
Posterior limb of the internal capsule	Michel J.M. et al^[11]^	Infarction of posterior limb of right internal capsule	1930s	Female	Tremor in both hands for nearly 10 yr	NA	Loss of left-sided tremor
	R. Duncan et al^[15]^	Infarction of the posterior limb of the left internal capsule	59	Male	Tremor in both hands for 1 yr	Yes	Loss of tremor in the right limb
Damage to the striatum	Shen-Yang Lim et al^[16]^	Lacunar infarction in the posterior part of the left putamen (striatum)	75	Male	Since the age of 10, there has been tremor affecting the hands, head, and voice	Yes	Improvement in right upper limb tremor, but not in head and voice tremor
	Rohan Mahale et al^[17]^	Left striatal envelope infarction	55	Male	Postural tremor of the hands since 20 yr of age	Yes	Improvement of right hand tremor
Thalamic infarction	Pradeep C. Bollu et al^[18]^	Bilateral thalamic and subthalamic infarcts	87	Female	Tremor with both hands	NA	Reduction of hand tremor
	A. Barbaud et al^[19]^	Left subthalamic lateral lacunar infarction	72	Female	Postural tremor of both upper limbs for 2 yr	No	Complete resolution of right upper limb tremor
Pontine infarction	Michel J.M. et al^[11]^	Left parapontine stroke	1925s	Male	Tremor of both upper limbs	Yes	Right-sided tremor completely disappeared
	N. Nagaratnam et al^[20]^	Infarction in the left pontine region	90	Male	Bilateral upper limb tremor persisting for almost 40 yr, worse on the right side than the left	Yes	Loss of right-sided tremor
Cerebellar infarction	M.J.M. Dupuis et al^[21]^	Abnormal MRI of the right cerebellar hemisphere, right cerebellar stroke	70	Male	Tremor in both hands since age 65, marked on the right side	Yes	Loss of right-sided tremor

ET = essential tremor, MRI = magnetic resonance imaging.

### 3.2. Case Analysis and literature review

The patient was hospitalized following an acute cerebral infarction in the right the right centrum semiovale of the brain, which was managed with thrombolytic therapy. Subsequent clinical evaluations and patient reports indicated a notable improvement in the patient’s original ET symptoms postinfarction. This paper seeks to analyze the potential neuroanatomical reasons for this outcome.

The site of cerebral infarction is located in the centrum semiovale, The centrum semiovale is situated in the white matter and comprises projection, association, and commissural fibers that link the cerebral cortex with subcortical regions. The thalamus serves as a crucial relay station for sensory, motor, and autonomic information to the cerebral cortex, with certain nuclei linked to the cerebral cortex via nerve fibers via the central semiovale area. Based on 2 imaging studies of this case, we localized and traced the infarct focus, the thalamus and 3 fiber conduction bundles crossing the infarct focus: the corticospinal tract (CST), thalamo-precentral gyrus fibers (T_PREC), and thalamo-postcentral gyrus fibers (T_POSTC).

In the pathogenesis of ET, abnormal neurotransmitter signals flow bidirectionally in the subcortex-cortex. This bi-directional flow indicates that subcortical areas, such as the thalamus, transmit signals to the cerebral cortex. At the same time, the cerebral cortex also conveys signals back to subcortical regions, forming an interactive loop.^[25]^ This loop may induce oscillations in the thalamocortical circuit, whereby neuronal activity between the thalamus and cerebral cortex synchronizes at a given frequency, resulting in oscillatory events. And the 3 nerve conduction fibers mentioned above that cross the infarct focus actually form a loop. The CST, being the most extensive collection of downstream nerve fibers, emanates from the somatomotor and somatosensory regions of the cerebral cortex and traverses the center of the semi-ovals and the posterior limb of the internal capsule to the spinal cord. The infarcted region in Figure 3 is located directly above the conduction pathway of the CST, disrupting the nerve conduction of the original CST. Ninety percent of the CST fibers in the cones split to form the lateral CST, which regulates the movement of the skeletal muscles in the limbs. Consequently, the infarction of the posterior limb of the internal capsule in both patients from the previous cases alleviated the tremor in the contralateral limb.^[11,15]^

T_PREC and T_POSTC are ascending fiber tracts that connect to the cortex via the thalamus. T_PREC terminates in the precentral gyrus via the thalamus. The premotor region of the cerebral cortex is crucial to bodily, limb, and facial movement, particularly in visually guided arm grabbing actions. The precentral gyrus represents the principal motor region of the cerebral cortex, in which axons from the ventral medial nucleus of the thalamus can activate particular neurons in the anterior-lateral motor cortex, thereby regulating motion and coordinating movements.^[26]^ Research has indicated that enhanced biological connection between the thalamic stimulation site and the motor cortex correlates with improved tremor suppression effects of deep brain stimulation for ET.^[27]^ T_POSTC terminates in the postcentral gyrus via the thalamus. Connections between the thalamus and the postcentral gyrus are essential for somatosensory processing. The ventral posterior lateral nucleus of the thalamus receives fibers from the spinal thalamic tract, constituting the majority of the thalamocortical tract. The thalamocortical tract culminates in the cortical sensory centers of the postcentral gyrus, which transmit sensations to the trunk and limbs. Figures 4 and 5 illustrate that the infarction focus is situated in the ascending nerve conduction tracts of these 2 nerves, hence impairing the conduction of the entire nerve circuit and interrupting the oscillation network that typically induces ET. A study using MRgFUS thalamotomy to target lesions in the ventral intermediate nucleus of the thalamus promotes functional recovery of symptom-related areas associated with sensorimotor and attentional networks,^[28]^ which also correlates with the function of T_PREC and T_POSTC. In the pathological state of ET, these 2 nerve conduction pathways may transmit abnormal neuroelectric signals, leading to motor and sensory impairments, notably tremor. It is worth noting that the infarcted area that interfered with nerve conduction bundles was the core infarcted area, and thus it was not affected by thrombolysis.

Before this, 2 other cases deviated from the right, 2 other cases deviated from the right semi-ovoid center yet nonetheless pertained to cerebral white matter infarction to enhance ET; the cases documented by F. Le Pira et al^[13]^ and Chunhua Xi et al^[14]^ both exhibited symptoms of left-sided corona radiata damage. The corona radiata is a component of the brain’s white matter, consisting of radial fibrous white matter extending from the internal capsule to the intercerebral cortex. It contains projection fibers traversing the center of the semiovals, along with nerve conduction bundles, including the corticomedullary tracts, CSTs, and anterior/posterior thalamic radiations. In both instances, the infarct symptoms manifested as numbness or hemiparesis of the right limb, and the recovery of the tremor was confined primarily to the right limb. The right central semiovale infarction in this patient exhibited bilateral improvement, suggesting that the infarction impacted more than merely the projection fibers. Previous studies indicating bilateral improvement in tremors are unusual. Joong-Seok Kim et al^[9]^ reported the resolution of tremor on the right side and significant improvement in the left hand and head of a patient with cortical infarction in the left precentral frontal area, offering a relevant reference for our case. We proposed that the right centrum semiovale infarction indirectly affected the corpus callosum. This conjecture was supported by visualizing the spatial relationship between the corpus callosum commissural fibers and the infarct foci (Fig. 6), where the infarction site was in close proximity to the right corpus callosum. Commissural fibers also penetrate the anterior centrum semiovale when projecting contralaterally. Although the infarct foci did not directly affect the core region of the corpus callosum, Wallerian degeneration may develop in adjacent callosal fibers, disrupting local white matter functional networks. The corpus callosum ensures precision of bimanual coordination by connecting bilateral motor cortices; its interhemispheric coupling may enable unilateral infarction to influence bilateral tremors through impaired interhemispheric information exchange and functional compensation, contributing to reduced cross-hemispheric oscillation synchronization.^[29]^ In a separate instance, the bilateral tremor improved due to the infarction focus being situated in the bilateral thalamus and subthalamus,^[18]^ so substantiating the thalamus’s critical function as a “relay station” within the ET oscillatory network. MRI gFUS thalamotomy is mostly conducted on the thalamus for drug-refractory ET.

## 4. Conclusion

In summary, the patient’s symptomatic improvement of ET after stroke, although incidental, can be explained by the ET pathogenesis-oscillatory network hypothesis centered around the cerebello-thalamo-cortical circuits as the intrinsic cause. The three-dimensional T1-weighted imaging, diffusion weighted imaging, and diffusion tensor imaging sequences of the MRI images were examined, and TractSeg was employed for enhanced nerve fiber tracking. Ultimately, BrainVoyager was employed to distinctly illustrate the spatial link among the lesion, thalamus, and associated fiber networks. The descending conduction of the corticospinal system and the 2 ascending conduction pathways from the thalamus to the cerebral cortex formed a complete neural circuit. The infarction’s location traversed the aforementioned conduction pathways and disrupted the oscillatory network. The findings from the literature review and this case demonstrate that the oscillatory network responsible for ET involves several critical nodes within the brain. Interference with a specific node may disrupt the oscillatory network of ET, potentially offering new avenues for future treatment strategies.

## Acknowledgments

We thank the patients and their caregivers for their involvement in the present study.

Supplemental Digital Content is available for this article (https://links.lww.com/MD/P525).

## Author contributions

**Data curation:** Xiaocheng Wang.

**Formal analysis:** Ruiyun Yu.

**Investigation:** Jingpei Wei, Chao Zhang, Haihuan Yang, Baoyun Qi.

**Methodology:** Dayong Ma, Yuanyuan Wang, Li Wang.

**Software:** Hua Zhang.

**Writing – original draft:** Linjing Song.

## Supplementary Material


