# Supplementary material for: Disappearance of bilateral essential tremor after acute stroke: A case report and literature review
Source: Medicine (Baltimore). 2026 Jan 23;105(4):e43537. doi: 10.1097/MD.0000000000043537 (PMC12851738; doi:10.1097/MD.0000000000043537)

Scale data sources: The questionnaire is a Chinese translated version of the Fahn–Tolosa–Marin Tremor Rating Scale, which was administered to the patient on 25 October 2024 at the time of his follow-up visit to the hospital.(Patient video as evidence,relevant videos can be submitted if necessary)

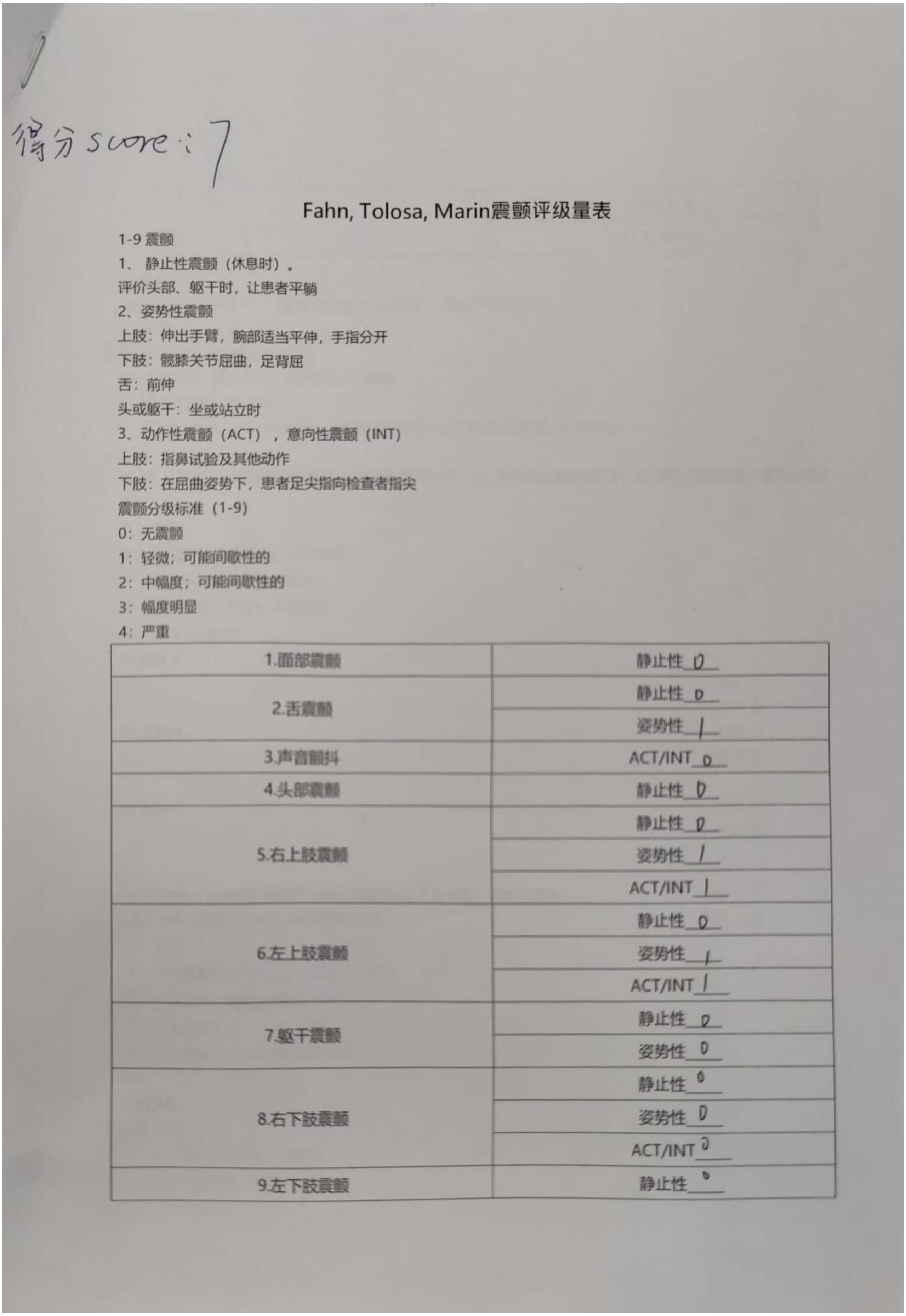

得分score：7

Fahn, Tolosa, Marin震颤评级量表

1-9 震颤

1、 静止性震颤（休息时）。

评价头部、躯干时，让患者平躺

2、姿势性震颤

上肢：伸出手臂，腕部适当平伸，手指分开

下肢：髋膝关节屈曲，足背屈

舌：前伸

头或躯干：坐或站立时

3、动作性震颤（ACT），意向性震颤（INT）

上肢：指鼻试验及其他动作

下肢：在屈曲姿势下，患者足尖指向检查者指尖

震颤分级标准（1-9）

0：无震颤

1：轻微；可能间歇性的

2：中幅度；可能间歇性的

3：幅度明显

4：严重

1.面部震颤	静止性 0
2.舌震颤	静止性 0
	姿势性 1
3.声音颤抖	ACT/INT 0
4.头部震颤	静止性 0
5.右上肢震颤	静止性 0
	姿势性 1
	ACT/INT 1
6.左上肢震颤	静止性 0
	姿势性 1
	ACT/INT 1
7.躯干震颤	静止性 0
	姿势性 0
8.右下肢震颤	静止性 0
	姿势性 0
	ACT/INT 0
9.左下肢震颤	静止性 0

	姿势性 0
	ACT/INT 0

10.书写

让患者书写标准句子："这是我的最佳书写样本，签名并写上日期。

0 = 正常

1 = 轻度异常。稍有邋遢，颤抖

2 = 中度异常。清晰可读，但有相当大的震颤。

3 = 标记为异常。难以辨认

4 = 严重异常。无法在不使用另一只手按住的情况下让铅笔或钢笔在纸上保持书写。

11-13. 让患者在不跨越线条的情况下连接各种图形的两个点。**先测试较弱的那只手，测试时手和胳膊都不要倚在桌子上。**

0 = 正常

1 = 稍有颤抖，可能会偶尔越过标线。

2 = 中度颤抖或频繁划错线条。

3 = 完成任务非常困难，错误众多。

4 = 无法完成绘图。

11.绘制 A

左手 0

右手 0

12.绘图 B

左手 0

右手 0

13.绘制 C

左手 0

右手 0

14. 使用约 *8* 厘米高的塑料杯，将水倒至距杯口 *1* 厘米处。让患者倒水，从一个杯子到另一个杯子，分别测试每只手。

左手 0

右手 0

0 = 正常

1 = 比无震颤的人更小心些，但水不会洒出.

2 = 少量溢水（最多为总量的 10%）.

3 = 会溢出大量水（> 10 - 50%）

4 = 无法倒水且大部分水都会洒出来.

15.口语

0 = 正常

1 = 仅"紧张"时声音轻微颤抖

2 = 轻度声音震颤，持续存在

3 = 中度声音震颤

4 = 严重的声音颤抖。说的有些话难以清楚理解.

16.除液体外的进食

0 = 正常

1 = 轻度正常。能够将所有固体食物送到嘴边，很少溢出。

2 = 中度异常。豌豆及类似食物频繁溢出。可能会把头至少伸到一半去接食物。

3 = 明显异常。无法用手进食。

4 = 严重异常。进食需要帮助。

17.将液体送至嘴边

0 = 正常

1 = 轻度异常。仍可使用勺子，但如果勺子完全装满则不行。

2 = 中度异常。无法用勺子；可使用杯子或玻璃杯

3 = 明显异常。能够用杯子或玻璃杯喝水，但需要双手。

4 = 严重异常。必须使用吸管。

18.进行卫生活动

0 = 正常

1 = 轻度异常。能够做所有事情，但比一般人更小心。

2 = 中度异常。能够做所有事情，但由于震颤而不能使用电动剃须刀

3 = 明显异常。无法完成大多数精细任务，如涂口红或刮胡子（即使使用电动剃须刀），除非使用双手。

4 = 严重异常。无法完成任何精细动作任务。

19.着装

0 = 正常

1 = 轻度异常。能够做所有事情，但比一般人更小心。

2 = 中度异常。能够做所有事情，但有错误。

3 = 明显异常。在扣扣子或其他活动（如系鞋带）方面需要一些帮助。

4 = 严重异常。即使进行粗大运动活动也需要协助。

20.写作

0 = 正常

1 = 轻度异常。书写清晰，可继续写信

2 = 中度异常。书写清晰，但不能再写信件

3 = 明显异常。难以辨认

4 = 严重异常。无法签名

21.工作

0 = 震颤不影响工作

1 = 能够工作，但需要比一般人更小心

2 = 能够做所有事情，但有错误。由于震颤，所以表现比往常差。

3 = 无法胜任日常工作。可能因震颤而换了一份不同的工作。震颤限制了家务劳动，比如熨衣服。

4 = 无法从事任何户外工作；能做的家务非常有限。

惯用手 左手 __ ; 右手 ✓

书写：这是我写得最好的一份样本。（This is a sample of my best handwriting ）

这是我写得最好的一份样本

签名：李风林

日期：10.25

绘制A、B 和C 图

DRAWING A

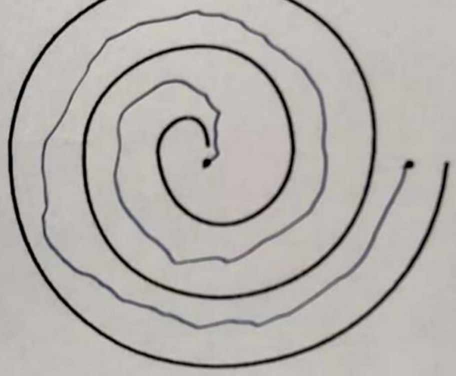

绘图 B

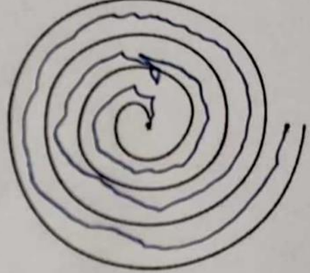

绘图 C

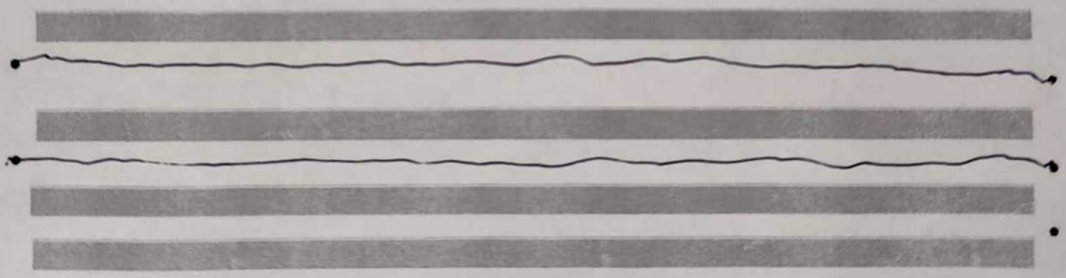

使用非惯用手 左手√；**右手**____

画出A、B 和C 图。

DRAWING A

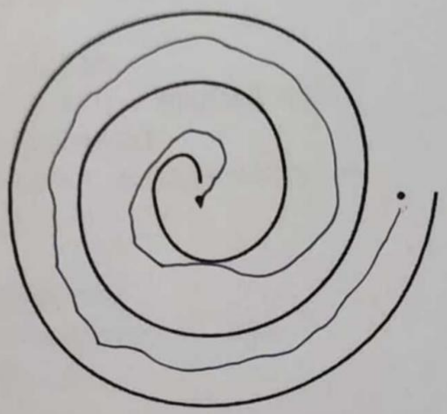

DRAWING B

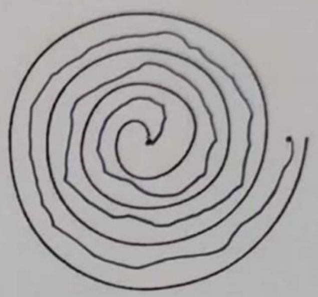

DRAWING C

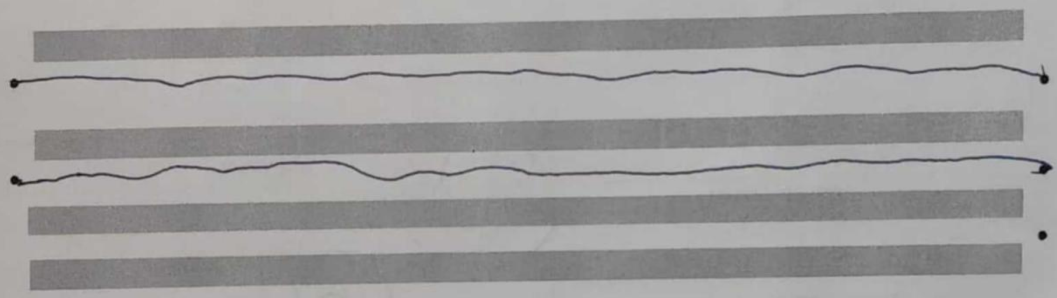

Supplement: Supplementary file 1 [file medi-105-e43537-s001.pdf]
